# Younger age, hyperextended knee, concomitant meniscectomy and large prerevision anterior tibial translation are associated with graft failure after the revision anterior cruciate ligament reconstruction

**DOI:** 10.1002/jeo2.70021

**Published:** 2024-09-25

**Authors:** Takeo Tokura, Takehiko Matsushita, Kyohei Nishida, Kanto Nagai, Noriyuki Kanzaki, Yuichi Hoshino, Tomoyuki Matsumoto, Ryosuke Kuroda

**Affiliations:** ^1^ Department of Orthopaedic Surgery Kobe University Graduate School of Medicine Kobe Japan

**Keywords:** anterior cruciate ligament, graft failure, revision surgery, space for the anterior cruciate ligament

## Abstract

**Purpose:**

Graft failure following revision anterior cruciate ligament (ACL) reconstruction is higher than after primary ACL reconstruction. However, data regarding revision surgery is scarce. We aimed to evaluate the associated factors for failure after revision ACL reconstruction.

**Methods:**

Fifty‐four patients (mean age: 24.7 ± 10.0 years) who underwent revision ACL reconstruction at our hospital with ≥1 year follow‐up were retrospectively examined. Patients were divided into Group F (graft failure) and Group N (no graft failure) groups. Univariate analysis was conducted to identify factors associated with graft failure. Receiver operating characteristic (ROC) curve analysis was performed to determine the optimal thresholds for differentiating between the two groups.

**Results:**

Graft failure was observed in 7 of 54 patients (13.0%). In the univariate analysis, significant differences were observed for age at the initial surgery (Group F: 15.6 ± 1.5, Group N: 20.9 ± 8.1), age at the revision surgery (Group F: 18.0 ± 2.8, Group N: 25.7 ± 10.3), presence of hyperextended knee (Group F: 85.7%, Group N: 14.9%), concomitant meniscectomy (Group F: 42.9%, Group N: 14.9%), prerevision space for the ACL (sACL) (Group F: 7.2 ± 3.4 mm, Group N: 13.4 ± 4.7 mm) and preoperative anterior tibial translation (ATT) (Group F: 5.0 ± 1.4 mm, Group N: 2.7 ± 3.1 m). ROC analysis of preoperative sACL and preoperative ATT on one‐leg standing plain radiograph showed that cutoff values of 6.9 and 4.2 mm were the optimal thresholds, respectively.

**Conclusion:**

Younger patients with a hyperextended knee, concomitant meniscectomy, small sACL and large ATT before revision ACL reconstruction are predisposed to graft failure.

**Level of Evidence:**

Level IV.

**Abbreviations**
ACL: anterior cruciate ligament
ATT: anterior tibial translation
BTB: bone–patellar tendon–bone
MRI: magnetic resonance imaging
PTS: posterior tibial slope
ROC: receiver operating characteristic
sACL: space for the anterior cruciate ligament

## INTRODUCTION

Anterior cruciate ligament (ACL) injuries are common knee injuries that often require ACL reconstruction. As the number of ACL reconstructions increases, the number of revision ACL surgeries also tend to rise [16]. In recent systematic reviews, graft failure frequency after revision surgery has been reported to be 6.0%–13.7% [6, 20, 38], which is nearly 3–4 times the primary ACL reconstructions [36, 37]. Clinical outcomes, including failure rate and patient‐reported outcomes, after revision surgery are reportedly worse than those after primary surgery [24, 34, 35]. Identifying risk factors for ACL revision surgery is a vital strategy for improving ACL revision outcomes in order to avoid more serious ACL injuries. However, there is a paucity of data on the risk factors for graft failure after revision ACL reconstruction.

A number of studies have reported the risk factors for graft failure after primary ACL reconstruction. Younger age, hyperextended knee, prerevision high‐grade pivot‐shift, steep tibial slope, anterior subluxation of tibia and meniscal injury have been suggested as risk factors in previous reports [9, 28, 29, 34]. Meanwhile, increased posterior tibial slope (PTS), medial meniscal injury and return to high‐risk sports are associated with graft failure or a third ACL injury after revision ACL reconstruction [8, 23, 35]. However, there are still a limited number of literature that have assessed the factors associated with failure after revision surgery.

Therefore, this study aimed to retrospectively explore the factors associated with graft failure after revision ACL reconstruction among patients with at least 1‐year follow‐up. The factors including patient background demographics, radiological factors and intraoperative factors, were compared between the groups with and without graft failure. It was hypothesized that some of the previously reported factors involved in graft failure following ACL reconstructions, such as hyperextended knee and large preoperative anterior tibial translation (ATT) on standing would be associated with greater risk of graft failure in revision surgery.

## MATERIALS AND METHODS

### Patient selection

All revision ACL reconstructions performed at our institution between January 2014 and June 2020 were reviewed retrospectively. Patients with at least 1‐year of follow‐up were included. Patients were excluded if they were lost to follow‐up before 1‐year follow‐up or had an additional osteotomy concomitant ligament injury requiring surgical intervention, a postsurgical infection, or incomplete data including imaging data or intraoperative findings.

**Figure 1:**
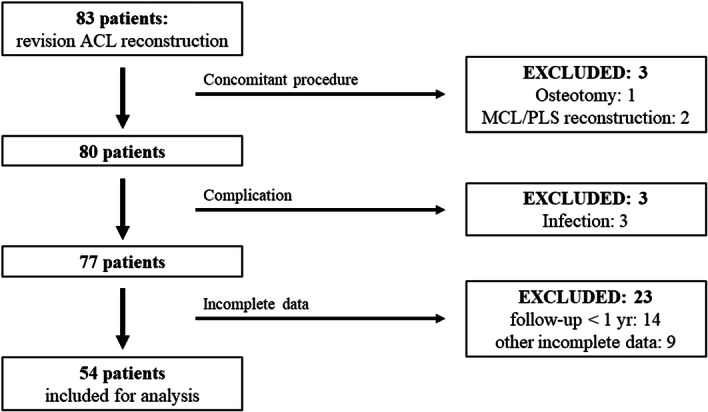
Flowchart of the patient selection process. ACL, anterior cruciate ligament; MCL, medial collateral ligament; PLS, poterolateral structures.

Originally, 83 patients were screened in this study. Of these, 54 patients (22 men and 32 women, mean age: 24.7 ± 10.0 years) fulfilled the inclusion criteria and were enroled in the present study (Figure 1). All the patients had routine follow‐up, including clinical assessment every 1–2 months and functional testing at sixth and 1 year, and annual visit after 1‐year follow‐up. Fourteen patients were lost to follow‐up before 1‐year postsurgery. All procedures performed in studies involving human participants were in accordance with the ethical standards of the institutional research committee of our institute, and the 1964 Helsinki Declaration and its later amendments or comparable ethical standards. This was a retrospective, observational study, and hence informed consent was not required from any participant, and the opt‐out method was applied based on the national law.

### Data Collection

The patient databases were searched for demographic, preoperative and postoperative data. Patient demographic data included age at the time of primary surgery, age at revision surgery, body mass index and sex. Data of prerevision surgery included the time from initial failure to revision surgery, Tegner activity scale, history of contralateral injury, presence of a hyperextended knee, preoperative pivot‐shift grade and preoperative KT‐1000 arthrometer (MED‐metric) measurement. Hyperextension of the knee was defined as an extension angle of >10° on physical examination according to the previous study [13]. Anterior knee laxity was evaluated by side‐to‐side differences in ATT using a KT‐1000 arthrometer at manual maximum load at 30° of knee flexion, which was measured immediately before surgery under general anaesthesia. According to the previous literature, interrater interclass correlation coefficient (ICC) ranged from 0.38 to 0.84 [26]. A pivot‐shift test was also performed under general anaesthesia before the revision surgery. An experienced orthopaedic surgeon performed the pivot‐shift test using a standardized technique [10]. The clinical grading was recorded according to International Knee Documentation Committee guidelines: none (−); glide (+); clunk (++); and gross (+++) [11]. Intraoperative data were obtained from the surgical records of revision surgeries, which included the presence of medial and lateral meniscal injuries and associated procedures (repair/meniscectomy), chondral injuries and grafts used for reconstruction. Graft failure was evaluated based on magnetic resonance imaging (MRI) findings and physical examination. It was diagnosed as graft failure when Lachman test was positive with no clear endpoint and pivot‐shift grade was 2 or 3 associated with the subjective symptoms of instability, or when imaging showed a new graft rupture [38]. Based on graft survival, patients with graft failure were allocated to Group F and those without graft failure were allocated to Group N. Data on return to sports was also collected at 1 year. If the patients were able to fully return to competition at any level, it was considered as being returned to sports.

### Radiographic evaluation

**Figure 2:**
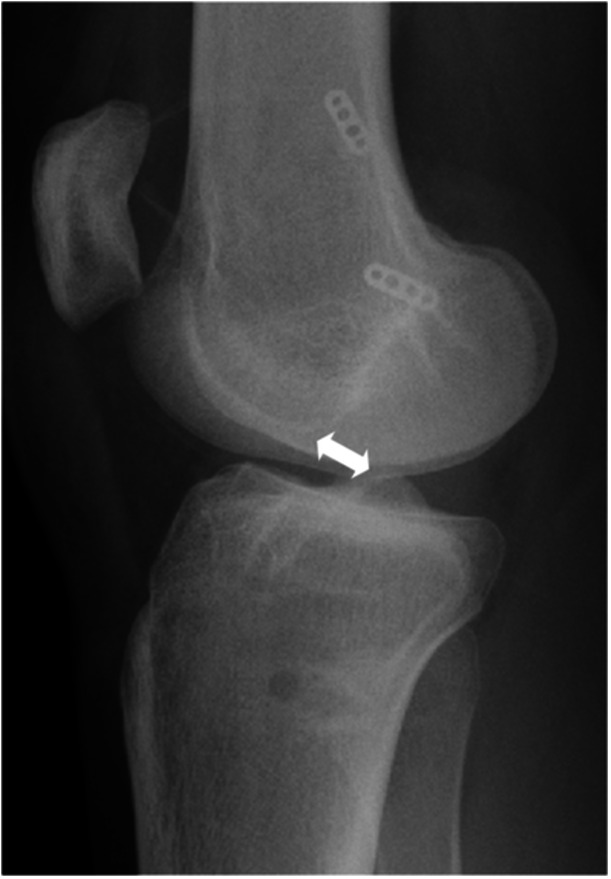
Radiographic measurements (space for the anterior cruciate ligament). Measurement of space for the anterior cruciate ligament. The distance between the tip of the tibial eminence and the most inferior portion of the Blumensaat line.

**Figure 3:**
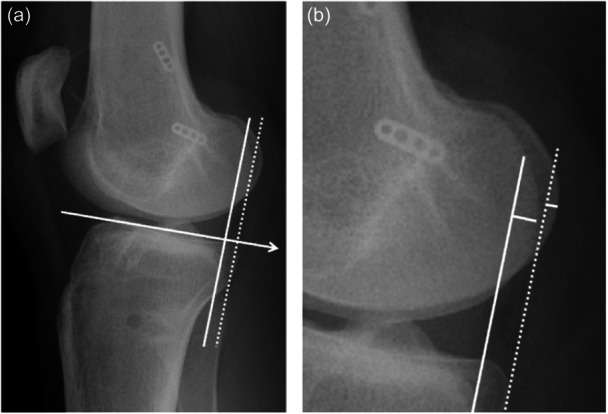
Radiographic measurements (anterior translation of tibia). (a) Anterior translation of the tibia measurement. A line was drawn along the subchondral plate of the tibial plateau (arrow). At the posterior margin of the medial and lateral tibial plateau, lines were drawn (solid line and dotted line) perpendicular to the first line (arrow). (b) The shortest distance from these lines to the most posterior cortical line of the femoral condyle was measured. Mean values for the medial and lateral sides were calculated.

All evaluations were performed on images taken after primary graft failure and before revision surgery. The ATT and space for the ACL (sACL) were evaluated using plain lateral‐view radiographs obtained during one‐leg standing position. For sACL, the distance between the tip of the tibial eminence and the most inferior portion of the Blumensaat line was measured as previously reported by Tanaka et al. [32] to evaluate potential impingement of the ACL graft due to tibial subluxation (Figure 2). ATT was measured as previously described [31]. The ATT was defined as the distance between the tangential line to the posterior tibial plateau and the posterior femoral condylar tangential line, which was parallel to the tibial plateau tangential line (Figure 3a,b). Radiographs with less than 2 mm separation difference between the posterior points of the medial and lateral femoral condyles were evaluated [31]. Measurements were performed on both the medial and lateral sides, and the mean values were recorded. According to the previous literature, interobserver ICCs were 0.85 for sACL and 0.94 for ATT [24, 31].

**Figure 4:**
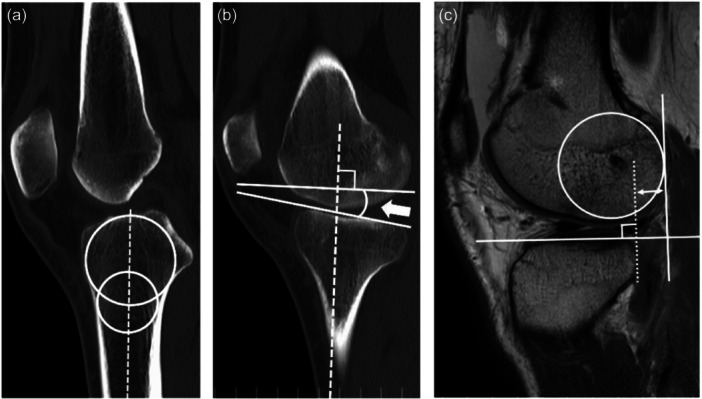
Measurement of posterior tibial slope and anterior translation of tibia (magnetic resonance imaging, computed tomography). (a, b) Measurement of posterior tibial slope (PTS). Two circles were drawn, which best fit in the proximal tibia. The centre of the caudal circle was positioned on the circumference of the cranial circle. Dotted line shows the tibial axis, which connects the centre of two circles and the arrow shows the PTS relative to the tibial axis. (c) Measurement of anterior tibial translation in magnetic resonance imaging and computed tomography. The line was drawn along the margin of the posterior margin of the tibia, which is perpendicular to the tibial plateau (dotted line). A best‐fit circle was drawn over the subchondral line of the posterior condyle, and a line perpendicular to the tibial plateau was then drawn along the posterior margin of the circle. The distance between the two lines was measured.

The PTS and ATT of both the medial and lateral plateaus were also measured using MRI and computed tomography as described in previous studies [22, 30] (Figure 4a–c). According to the previous studies, interobserver ICC was 0.80 for PTS, 0.72 for medial ATT and 0.96 for lateral ATT [22, 30]. All measurements were performed by a single examiner who was blinded to graft survival.

### Surgical procedure

After primary ACL graft failure was diagnosed based on physical examination and radiological evaluation, revision ACL reconstruction was scheduled and performed using an anatomical reconstruction technique. In the primary reconstruction, bone–patellar tendon–bone (BTB), artificial ligament or hamstring tendon was used based on surgeons' preference. BTB or hamstring tendon was used for revision surgery depending on the surgeon's preference. Either meniscectomy or meniscus repair was performed for meniscus injuries depending on the surgeons' decision. A suspensory fixation device or interference screw was used for the femoral side and a post screw was used for the tibial side. If a tibial tunnel enlargement was observed, an interference screw was added for tibial fixation.

### Postoperative rehabilitation

A standardized postoperative rehabilitation protocol was applied to all patients, with some modification on weight‐bearing based on meniscal status. Progressive range of motion exercises and one‐third weight bearing on the operated limb were started 3 days after surgery, and full weight bearing was allowed 2 weeks after surgery. An ACL brace (DON‐JOY FULLFORCE; DJO) was worn for 2 months postoperatively. Jogging was permitted 3 months postoperatively, followed by a gradual progression of endurance and agility exercises. Full return to sports was permitted approximately 9 months after surgery, taking into account the patients' muscle strength and functional recovery.

### Statistical analyses

All statistical analyses of the recorded data were performed using the Excel statistical software package Bell Curve for Excel (Social Survey Research Information Co., Ltd.). Statistical significance was defined as *p* < 0.05. Fisher's exact test and the Mann–Whitney *U* test were used to compare factors between the two groups. For the preoperative sACL and ATT on one‐leg standing plain radiographs, receiver operating characteristic (ROC) curve analysis was used to identify the optimal cutoff point for graft failure between the two groups. The point on the curve closest to the (0, 1) point was used to identify the optimal cut‐off point [1]. The results of each data were expressed in median and interquartile range unless otherwise described. A priori power analysis was performed by G*Power 3.1.9.4 (Franz Paul). For analysis of sACL, in order to detect a 3.5 mm difference, which was defined as a cutoff value for the risk factor of graft failure in chronic cases [32], and assuming 10% of the failure rate, a total of at least 34 patients were needed to achieve power 0.8. For categorical data, if the effect size was set at 0.3, 88 patients were needed.

## RESULTS

The types of injuries associated with the initial ACL tear and graft failure after primary ACL reconstruction are shown in Table 1. Indirect contact injuries involve injuries that result from the patients' own movements, but are disturbed by a physical perturbation, whereas direct injuries involve injuries resulted from direct force applied around the knee [19]. Primary injuries were sports‐related in 85% of the patients, and secondary injuries were 74% sports‐related. The average follow‐up period was 33.0 ± 23.7 months (range: 12–142 months). Seven out of 54 patients (13.0%) suffered from further graft failure (third injury) after revision surgery. Among these patients, four had atraumatic failure, without a clear episode of injury, two had a noncontact injury and one had a direct contact injury. The percentage of the patients who had returned to sports at one year postoperatively was 53.7% (29/54 patients). Of 29 patients who returned to sports at 1 year, three patients had graft failure.

**Table 1:** Injury pattern.

Initial ACL tear	
Noncontact	38 (70.4%)
Direct contact	5 (9.3%)
Indirect contact	3 (5.6%)
Biological failure	0 (0%)
Not available	8 (14.8%)
Second injury	
Noncontact	43 (79.6%)
Direct contact	0 (0%)
Indirect contact	5 (9.3%)
Biological failure	6 (11.1%)

Abbreviation: ACL, anterior cruciate ligament.

In the primary ACL reconstruction, BTB was used in one patient, artificial ligament in two patients and hamstring tendon in 46 patients (19 were single‐bundle and 27 were double‐bundle). There were no data available about the graft choice for the primary ACL reconstruction in five patients. In the revision surgery, BTB was used in 51 patients and hamstring tendons in three patients. At the time of primary ACL reconstructions, four patients received meniscectomy, and 13 patients received meniscal repair. Data were not available in 12 patients. Single‐staged surgeries were performed in 52 patients and one patient from each group underwent two‐staged surgery. With regards to the femoral fixation for revision surgeries, 27 patients (50%) had suspensory fixation, 16 (29.6%) had interference screw fixation and 4 (7.4%) had both suspensory and interference screw fixation. For the tibial fixation, 11 (20.4%) patients had post screw fixation, 26 (48.1%) had interference screw fixation and 10 (18.5%) had both post screw and interference fixation. Seven patients (13%) were lacking the data about the fixation methods. Two patients in Group N had additional lateral extra‐articular tenodesis at the time of revision surgeries, and one had anterolateral ligament reconstruction in Group F.

Data about patient demographics, intraoperative findings and radiographic measurements of the total cohort are shown in Table 2. Seven patients in Group N and two patients in Group F had open physes at the time of primary ACL reconstruction, which was not statistically different. None of the patients had past ipsilateral injuries, except for ACL injuries.

**Table 2:** Patient demographics, preoperative data, radiographic measurements.^a^

	Total cohort (*n* = 54)
Patient demographics	
Sex, male/female	22/32
Age at initial surgery, years	17.5 (16.0–21.8)
Age at revision surgery, years	21.0 (18.0–27.0)
Tegner activity scale	7 (6.3–9.0)
Period from injury to revision surgery, months	2.0 (1.0–6.0)
BMI (kg/m^2^)	21.1 (20.4–23.0)
Hyperextended knee, *n* (%)	13 (24.1)
Contralateral injury, *n* (%)	7 (13.0)
Preop pivot‐shift test grade, grade 0, 1/grade 2, 3	18/36
Preop KT‐1000 SSD (mm)	6.0 (5.0–8.0)
Intraoperative findings	
Medial meniscus injury, *n* (%)	27 (50.0)
Lateral meniscus injury, *n* (%)	19 (35.1)
Meniscectomy, *n* (%)	10 (18.5)
Meniscal repair, *n* (%)	25 (46.3)
Chondral injury, *n* (%)	17 (31.5)
Revision graft, BTB/hamstrings	51/3
Preoperative measurements	
sACL (mm)	11.5 (9.9–14.5)
ATT (mm)	3.2 (2.0–4.8)
Medial PTS (°)	6.3 (4.6–8.2)
Lateral PTS (°)	6.9 (5.0–8.1)
Medial ATT CT (mm)	1.8 (0–3.4)
Lateral ATT CT (mm)	0.4 (−2.7 to 2.7)
Medial ATT MRI (mm)	1.8 (0–4.4)
Lateral ATT MRI (mm)	4.1 (1.4–7.4)

Abbreviations: ATT, anterior tibial translation; BMI, body mass index; BTB, bone–patellar tendon–bone; CT, computed tomography; MRI, magnetic resonance image; ns, not significant; PTS, posterior tibial slope; sACL, space for the anterior cruciate ligament; SSD, side‐to‐side difference.

^a^ Data reported as median (interquartile range) unless otherwise indicated. Meniscal and chondral injuries were confirmed via arthroscopic inspection during surgery. ‘Preoperative’ refers to before revision surgery unless otherwise indicated.

Univariate analyses showed that the age at the time of primary and revision surgery, the ratio of the presence of a hyperextended knee and concomitant meniscectomy and prerevision sACL and ATT in group F were significantly younger, higher and larger, respectively, than those in group N (Table 3). The results of the post hoc power analyses are shown in Table 4.

**Table 3:** Patient demographics, preoperative data, radiographic measurements.^a^

	Group N (*n* = 47)	Group F (*n* = 7)	*p* Value
Patient demographics			
Sex, male/female	21/26	1/6	ns
Age at initial surgery, years	19 (16.0–22.5)	15 (14.5–16.5)	0.019*
Age at revision surgery, years	23.0 (18.5–28.0)	18.0 (15.5–20.0)	0.015*
Tegner activity scale	7.0 (6.5–9.0)	9.0 (6.0–9.5)	ns
Period from injury to revision surgery, months	2.0 (1.0–6.0)	1.0 (1.0–5.0)	ns
BMI (kg/m^2^)	21.0 (20.3–22.5)	22.8 (20.6–23.1)	ns
Hyperextended knee, *n* (%)	7 (14.9)	6 (85.7)	<0.001*
Contralateral injury, *n* (%)	5 (10.6)	2 (28.6)	ns
Preop pivot‐shift test grade, grade 0, 1/grade 2, 3	16/31	2/5	ns
Preop KT‐1000 SSD (mm)	6.0 (4.0–8.0)	8.5 (6.0–11.8)	ns
Intraoperative findings			
Medial meniscus injury, *n* (%)	22 (46.8)	5 (71.4)	ns
Lateral meniscus injury, *n* (%)	17 (36.2)	2 (28.6)	ns
Meniscectomy, *n* (%)	7 (14.9)	3 (42.9)	0.0022*
Meniscal repair, *n* (%)	23 (48.9)	2 (28.6)	ns
Chondral injury, *n* (%)	14 (30.0)	3 (42.9)	ns
Revision graft, BTB/hamstrings	44/3	7/0	ns
Lateral extra‐articular augmentation, *n* (%)	2 (4.3)	1 (14.2)	ns
Preoperative measurements			
sACL (mm)	11.9 (10.2–15.0)	6.4 (5.8–9.8)	0.0035*
ATT (mm)	3.0 (1.7–4.6)	4.8 (4.3–5.8)	0.031*
Medial PTS (°)	7.1 (4.7–8.3)	5.8 (2.1–6.2)	ns
Lateral PTS (°)	6.9 (5.0–8.3)	8.0 (4.7–8.0)	ns
Medial ATT CT (mm)	1.9 (0–3.5)	1.2(0.3–1.8)	ns
Lateral ATT CT (mm)	−0.7 (−2.5 to 2.4)	2.4 (−1.9 to 3.6)	ns
Medial ATT MRI (mm)	1.7 (0–3.6)	3.9 (2.3–5.0)	ns
Lateral ATT MRI (mm)	3.7 (1.3–7.2)	7.6 (4.8–10.2)	ns

Abbreviations: ATT, anterior tibial translation; BMI, body mass index; BTB, bone–patellar tendon–bone; CT, computed tomography; MRI, magnetic resonance image; ns, not significant; PTS, posterior tibial slope; sACL, space for the anterior cruciate ligament; SSD, side‐to‐side difference.

^a^ Data reported as median (interquartile range) unless otherwise indicated. Meniscal and chondral injuries were confirmed via arthroscopic inspection during surgery. ‘Preoperative’ refers to before revision surgery unless otherwise indicated. Lateral extra‐articular augmentation includes lateral extra‐articular tenodesis and anterolateral ligament reconstruction.

* Statistical significance: *p* < 0.05.

**Table 4:** Post hoc power analysis.^a^

	Pot hoc power (1 − *β*)
Patient demographics	
Sex, male/female	0.30
Age at initial surgery, years	0.70
Age at revision surgery, years	0.78
Tegner activity scale	0.070
Period from injury to revision surgery, months	0.10
BMI (kg/m^2^)	0.11
Hyperextended knee, *n* (%)	0.98
Contralateral injury, *n* (%)	0.21
Preop pivot‐shift test grade, grades 0, 1/grades 2, 3	0.030
Preop KT‐1000 SSD (mm)	0.64
Intraoperative findings	
Medial meniscus injury, *n* (%)	0.26
Lateral meniscus injury, *n* (%)	0.042
Meniscectomy, *n* (%)	0.36
Meniscal repair, *n* (%)	0.13
Chondral injury, *n* (%)	0.098
Revision graft, BTB/hamstrings	1.0
Lateral extra‐articular augmentation, *n* (%)	0.14
Preoperative measurements	
sACL (mm)	0.97
ATT (mm)	0.73
Medial PTS (°)	0.21
Lateral PTS (°)	0.072
Medial ATT CT (mm)	0.098
Lateral ATT CT (mm)	0.25
Medial ATT MRI (mm)	0.37
Lateral ATT MRI (mm)	0.60

Abbreviations: ATT, anterior tibial translation; BMI, body mass index; BTB, bone–patellar tendon–bone; CT, computed tomography; MRI, magnetic resonance image; PTS, posterior tibial slope; sACL, space for the anterior cruciate ligament; SSD, side‐to‐side difference.

^a^ Meniscal and chondral injuries were confirmed via arthroscopic inspection during surgery. ‘Preoperative’ refers to before revision surgery unless otherwise indicated. Lateral extra‐articular augmentation includes lateral extra‐articular tenodesis and anterolateral ligament reconstruction.

**Figure 5:**
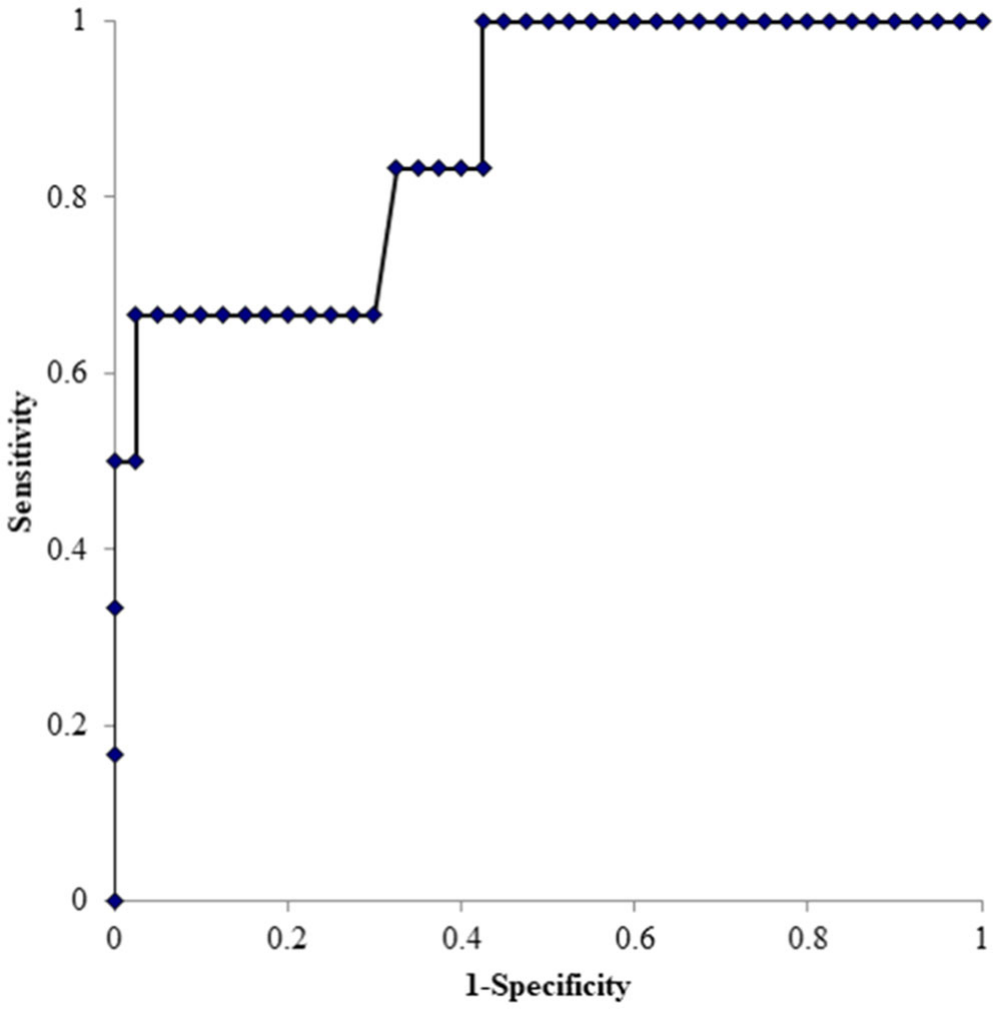
ROC analysis of space for the anterior cruciate ligament. The area under the ROC curve was 0.87 (95% CI, 0.71–1.03: *p* < 0.01). The cutoff point value was 6.9 mm: sensitivity, 67%, specificity 98%. CI, confidence interval; ROC, receiver operating characteristics.

**Figure 6:**
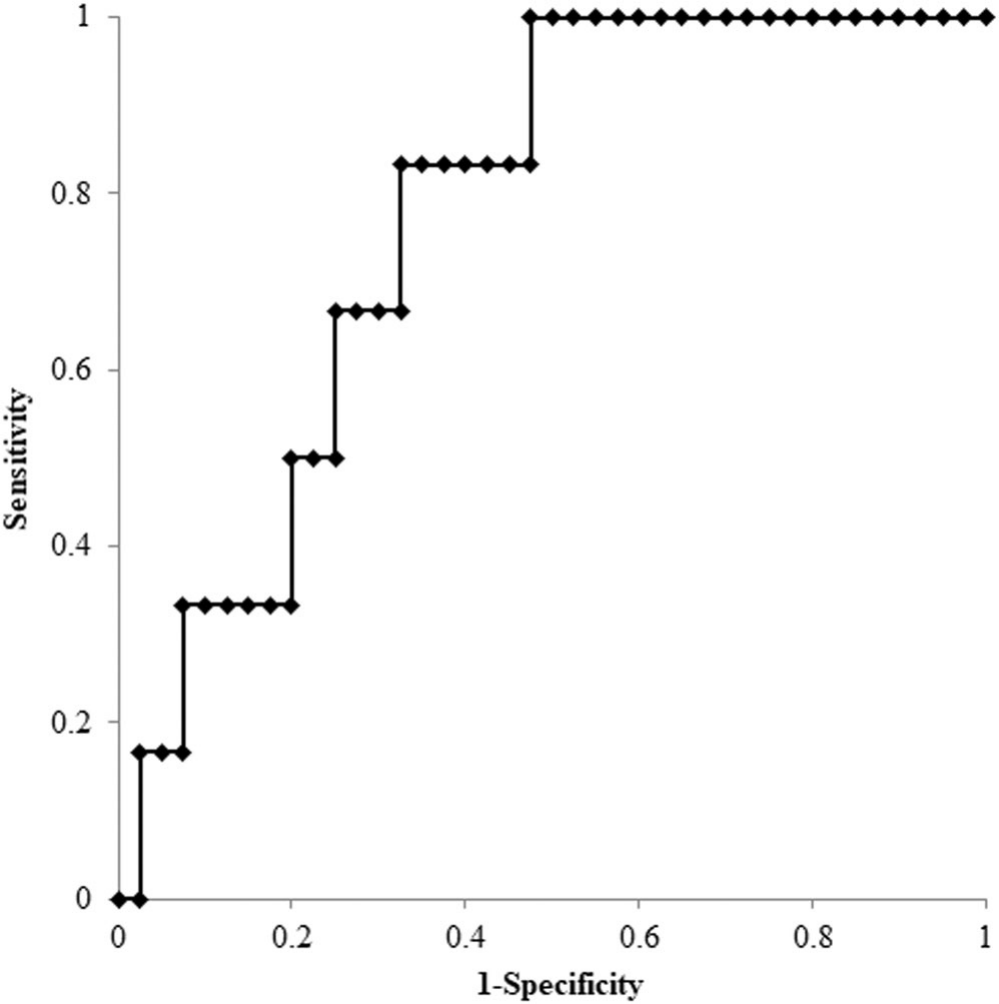
ROC analysis of anterior tibial translation on one‐leg standing plain radiograph. The area under the ROC curve was 0.78 (95% CI, 0.61–0.94: *p* < 0.001). The cutoff point value was 4.2 mm: sensitivity 83%, specificity 68%. CI, confidence interval; ROC, receiver operating characteristics.

For the prerevision sACL, ROC curve analysis determined a cutoff value of 6.9 mm as the optimal threshold for differentiating between the two groups (area under curve: 0.87, 95% confidence interval [CI]: 0.71–1.03, sensitivity: 67%, specificity: 98%), while for the ATT on one‐leg standing plain radiograph, 4.2 mm was determined as the optimal threshold (area under curve: 0.78, 95% CI: 0.61–0.94, sensitivity: 83%, specificity: 68%) (Figures 5 and 6).

## DISCUSSION

The main findings of the present study were that younger age, hyperextended knee, concomitant meniscectomy, small prerevision sACL and large prerevision ATT on lateral‐view radiographs were associated with graft failure after revision ACL reconstruction.

Previous studies have reported that young age is a risk factor for graft failure after primary ACL reconstruction [3, 39]. In the present study, the mean age of patients in the graft failure group was lower than that of patients in the nonfailure group. The reasons for this result are likely multifactorial. One explanation for this is that younger patients tend to return to original sports level and have less fear for reinjury, which may increase the risk of graft failure [3, 5, 39]. Webster et al. [35] reported that in patients aged < 25 years, the presence of medial meniscal pathology and return to high‐risk sports were significantly associated with a third ACL injury. Although there was no statistically significant difference in Tegner Activity Scale between the graft failure group and no graft failure group, it was higher in the graft failure group. Therefore, the pattern of activity can partially account for high graft failure rates in young patients. Physeal status (closed or open) may be another factor, but in the present study, it was not associated with the graft failure. In the present study, the presence of a hyperextended knee was also identified as a significant factor associated with graft failure. Previous literatures showed that there are higher forces generated in the ACL graft during knee hyperextension, and that might attribute to ACL graft stretching and failure [17, 18]. This is also consistent with previous clinical studies that reported hyperextend knee was a risk factor for graft failure and residual pivot‐shift phenomenon [13, 15]. Therefore, the surgeons must be aware of these results when performing revision ACL reconstruction on young and active patients with hyperextended knees.

Concomitant meniscectomy was also associated with graft failure in the present study. Menisci function as secondary restraint of the knee, thus meniscal deficiency could lead to increased knee instability and stress to the ACL graft, which may eventually result in increased graft failure [21]. Parkinson et al. showed in their study that the hazard ratio of medial and lateral meniscal deficiency for graft failure was 15.1 and 9.9, respectively, in the primary ACL reconstruction [25]. Webster et al. also showed that medial meniscal pathology was one of the factors associated with higher multiple ACL injury rate [35]. These results suggest that it is important to preserve menisci in ACL reconstruction to prevent graft failure, although menisci may be no longer repairable after multiple ACL injuries especially in the revision setting.

In the present study, the sACL was identified as a factor associated with graft failure after ACL revision. Tanaka et al. [32] examined the influence of anterior tibial subluxation on early graft failure without trauma and demonstrated that a small preoperative sACL, which was measured on lateral‐view radiographs to estimate the space of the reconstructed ACL in passive maximum extension, was the most significant risk factor for early graft failure after primary ACL reconstruction in chronic ACL‐deficient knees. In revision ACL, excessive anterior tibial subluxation due to recurrent knee laxity after primary ACL or inherently greater knee laxity can be present at the time of surgery [8], which makes those cases somewhat similar to chronic ACL‐deficient knees. Therefore, anterior subluxation may be a risk factor in revision setting as well. In the previous study, the sACL correlated with the ATT on plain radiographs, which has often been used to evaluate anterior tibial subluxation [2, 7]. Although the sACL was originally measured using plain lateral radiographs taken in the supine position, plain radiographs taken in the one‐leg standing position were used in the present study to mimic knee status more closely during standing and walking. It has been shown that ATT in knee extension increases under the weight‐bearing condition in the ACL‐deficient knees [14, 33]. Moreover, there is a greater joint compressive load and quadriceps muscle force in the standing state, which could exacerbate the anterior translation of the tibia [33]. The results of the present study suggest that prerevision sACL and ATT on lateral‐view plain radiographs taken during single‐leg standing position could be useful in predicting graft failure after revision ACL reconstruction.

The present study has several limitations. First, a small number of patients experienced graft failure, which might have resulted in an underpowered analysis of each risk factor, and multivariate analysis could not be performed for this reason. However, the revision surgeries are not frequently performed, unlike primary reconstructions; around 20 cases each year at our institution, and the failure rate is approximately 10%, which makes it difficult to enrol large number of patients with third ACL injury. Second, technical errors, such as tunnel malposition, were not assessed in this study. There were several studies showing that the technical errors were the most common cause of the graft failure [12, 27]. The surgeries in the present study were performed by experienced surgeons using a uniform strategy to minimize technical errors. However, this may have affected the results of the present study. Third, the minimum follow‐up period for the present study was 1 year, which might be too short to assess graft failures.

Despite these limitations, this study investigated the risk factors for graft failure after revision ACL reconstruction and provided important information to consider when managing these patients.

## CONCLUSIONS

Younger age, hyperextended knee, concomitant meniscectomy, small prerevision sACL and large prerevision ATT were associated with graft failure after revision ACL reconstruction.

## AUTHOR CONTRIBUTIONS

Takeo Tokura was involved in the conception and design of the study, the acquisition, analysis and interpretation of the data and writing the article. Takehiko Matsushita was involved in the conception and design of the study, development of the research and writing the article. Kyohei Nishida, Kanto Nagai, Noriyuki Kanzaki, Yuichi Hoshino, Tomoyuki Matsumoto and Ryosuke Kuroda were involved in the acquisition and interpretation of the data. All of the authors were involved in the critical revisions of the article for its important intellectual content, and they all approved the final version of the article.

## CONFLICT OF INTEREST STATEMENT

The authors declare no conflicts of interest.

## ETHICS STATEMENT

All procedures performed in studies involving human participants were in accordance with the ethical standards of the institutional research committee (the Institutional Review Board of Kobe University (ID No. B190055), and the 1964 Helsinki Declaration and its later amendments or comparable ethical standards.

## Data Availability

The data that support the findings of this study are available from the corresponding author upon reasonable request.

## References

[1] Akobeng, A.K. (2007) Understanding diagnostic tests 3: receiver operating characteristic curves. Acta Paediatrica, 96(5), 644–647. Available from: 10.1111/j.1651-2227.2006.00178.x 17376185

[2] Almekinders, L.C. & de Castro, D. (2001) Fixed tibial subluxation after successful anterior cruciate ligament reconstruction. The American Journal of Sports Medicine, 29(3), 280–283. Available from: 10.1177/03635465010290030301 11394594

[3] Astur, D.C. , Cachoeira, C.M. , da Silva Vieira, T. , Debieux, P. , Kaleka, C.C. & Cohen, M. (2018) Increased incidence of anterior cruciate ligament revision surgery in paediatric verses adult population. Knee Surgery, Sports Traumatology, Arthroscopy, 26(5), 1362–1366. Available from: 10.1007/s00167-017-4727-z 28948312

[4] Berry, J. , Kramer, K. , Binkley, J. , Binkley, G.A. , Stratford, P. , Hunter, S. et al. (1999) Error estimates in novice and expert raters for the KT‐1000 arthrometer. Journal of Orthopaedic & Sports Physical Therapy, 29(1), 49–55. Available from: 10.2519/jospt.1999.29.1.49 10100121

[5] Chicorelli, A.M. , Micheli, L.J. , Kelly, M. , Zurakowski, D. & MacDougall, R. (2016) Return to sport after anterior cruciate ligament reconstruction in the skeletally immature athlete. Clinical Journal of Sport Medicine, 26(4), 266–271. Available from: 10.1097/JSM.0000000000000275 27359295

[6] D'Ambrosi, R. , Meena, A. , Raj, A. , Ursino, N. , Formica, M. , Herbort, M. et al. (2023) Multiple revision anterior cruciate ligament reconstruction: not the best but still good. Knee Surgery, Sports Traumatology, Arthroscopy, 31(2), 559–571. Available from: 10.1007/s00167-022-07197-8 PMC9898374 36224291

[7] Franklin, J.L. , Rosenberg, T.D. , Paulos, L.E. & France, E.P. (1991) Radiographic assessment of instability of the knee due to rupture of the anterior cruciate ligament. A quadriceps‐contraction technique. The Journal of Bone & Joint Surgery, 73(3), 365–372. Available from: 10.2106/00004623-199173030-00007 2002074

[8] Grassi, A. , Macchiarola, L. , Urrizola Barrientos, F. , Zicaro, J.P. , Costa Paz, M. , Adravanti, P. et al. (2019) Steep posterior tibial slope, anterior tibial subluxation, deep posterior lateral femoral condyle, and meniscal deficiency are common findings in multiple anterior cruciate ligament failures: an MRI case‐control study. The American Journal of Sports Medicine, 47(2), 285–295. Available from: 10.1177/0363546518823544 30657705

[9] Guimarães, T.M. , Giglio, P.N. , Sobrado, M.F. , Bonadio, M.B. , Gobbi, R.G. , Pécora, J.R. et al. (2021) Knee hyperextension greater than 5° is a risk factor for failure in ACL reconstruction using hamstring graft. Orthopaedic Journal of Sports Medicine, 9(11), 232596712110563. Available from: 10.1177/23259671211056325 PMC8606942 34820464

[10] Hoshino, Y. , Araujo, P. , Ahlden, M. , Moore, C.G. , Kuroda, R. , Zaffagnini, S. et al. (2012) Standardized pivot shift test improves measurement accuracy. Knee Surgery, Sports Traumatology, Arthroscopy, 20(4), 732–736. Available from: 10.1007/s00167-011-1850-0 22205096

[11] Irrgang, J.J. , Ho, H. , Harner, C.D. & Fu, F.H. (1998) Use of the International Knee Documentation Committee guidelines to assess outcome following anterior cruciate ligament reconstruction. Knee Surgery, Sports Traumatology, Arthroscopy, 6(2), 107–114. Available from: 10.1007/s001670050082 9604196

[12] Johnson, D.L. & Fu, F.H. (1995) Anterior cruciate ligament reconstruction: why do failures occur? Instructional Course Lectures, 44, 391–406. 7797878

[13] Kamada, K. , Matsushita, T. , Nagai, K. , Hoshino, Y. , Araki, D. , Kanzaki, N. et al. (2022) Risk factors of residual pivot‐shift after anatomic double‐bundle anterior cruciate ligament reconstruction. Archives of Orthopaedic and Trauma Surgery, 143(2), 977–985. Available from: 10.1007/s00402-022-04428-y 35364734

[14] Li, G. , Rudy, T.W. , Allen, C. , Sakane, M. & Woo, S.L.Y. (1998) Effect of combined axial compressive and anterior tibial loads on in situ forces in the anterior cruciate ligament: a porcine study. Journal of Orthopaedic Research, 16(1), 122–127. Available from: 10.1002/jor.1100160121 9565084

[15] Lindanger, L. , Strand, T. , Mølster, A.O. , Solheim, E. & Inderhaug, E. (2021) Effect of early residual laxity after anterior cruciate ligament reconstruction on long‐term laxity, graft failure, return to sports, and subjective outcome at 25 years. The American Journal of Sports Medicine, 49(5), 1227–1235. Available from: 10.1177/0363546521990801 33656379

[16] Lynch, T.S. , Parker, R.D. , Patel, R.M. , Andrish, J.T. & Spindler, K.P. (2015) The impact of the Multicenter Orthopaedic Outcomes Network (MOON) research on anterior cruciate ligament reconstruction and orthopaedic practice. Journal of the American Academy of Orthopaedic Surgeons, 23(3), 154–163. Available from: 10.5435/JAAOS-D-14-00005 25667401 PMC4344406

[17] Markolf, K.L. , Gorek, J.F. , Kabo, J.M. & Shapiro, M.S. (1990) Direct measurement of resultant forces in the anterior cruciate ligament. An in vitro study performed with a new experimental technique. The Journal of Bone & Joint Surgery, 72(4), 557–567. Available from: 10.2106/00004623-199072040-00014 2324143

[18] Markolf, K.L. , Burchfield, D.M. , Shapiro, M.M. , Shepard, M.F. , Finerman, G.A.M. & Slauterbeck, J.L. (1995) Combined knee loading states that generate high anterior cruciate ligament forces. Journal of Orthopaedic Research, 13(6), 930–935. Available from: 10.1002/jor.1100130618 8544031

[19] Marshall, S.W. (2010) Recommendations for defining and classifying anterior cruciate ligament injuries in epidemiologic studies. Journal of Athletic Training, 45(5), 516–518. Available from: 10.4085/1062-6050-45.5.516 20831401 PMC2938327

[20] Mohan, R. , Webster, K.E. , Johnson, N.R. , Stuart, M.J. , Hewett, T.E. & Krych, A.J. (2018) Clinical outcomes in revision anterior cruciate ligament reconstruction: a meta‐analysis. Arthroscopy: The Journal of Arthroscopic & Related Surgery, 34(1), 289–300. Available from: 10.1016/j.arthro.2017.06.029 28866344

[21] Musahl, V. , Citak, M. , O'Loughlin, P.F. , Choi, D. , Bedi, A. & Pearle, A.D. (2010) The effect of medial versus lateral meniscectomy on the stability of the anterior cruciate ligament‐deficient knee. The American Journal of Sports Medicine, 38(8), 1591–1597. Available from: 10.1177/0363546510364402 20530720

[22] Nagai, K. , Tashiro, Y. , Herbst, E. , Gale, T. , Wang, J.H. , Irrgang, J.J. et al. (2018) Steeper posterior tibial slope correlates with greater tibial tunnel widening after anterior cruciate ligament reconstruction. Knee Surgery, Sports Traumatology, Arthroscopy, 26(12), 3717–3723. Available from: 10.1007/s00167-018-5004-5 29869200

[23] Napier, R.J. , Garcia, E. , Devitt, B.M. , Feller, J.A. & Webster, K.E. (2019) Increased radiographic posterior tibial slope is associated with subsequent injury following revision anterior cruciate ligament reconstruction. Orthopaedic Journal of Sports Medicine, 7(11), 2325967119879373. Available from: 10.1177/2325967119879373 31723566 PMC6831974

[24] Ni, Q. , Song, G. , Zhang, Z. , Zheng, T. , Feng, Z. , Cao, Y. et al. (2020) Steep posterior tibial slope and excessive anterior tibial translation are predictive risk factors of primary anterior cruciate ligament reconstruction failure: a case‐control study with prospectively collected data. The American Journal of Sports Medicine, 48(12), 2954–2961. Available from: 10.1177/0363546520949212 32866043

[25] Parkinson, B. , Robb, C. , Thomas, M. , Thompson, P. & Spalding, T. (2017) Factors that predict failure in anatomic single‐bundle anterior cruciate ligament reconstruction. The American Journal of Sports Medicine, 45(7), 1529–1536. Available from: 10.1177/0363546517691961 28296429

[26] Pugh, L. , Mascarenhas, R. , Arneja, S. , Chin, P.Y.K. & Leith, J.M. (2009) Current concepts in instrumented knee‐laxity testing. The American Journal of Sports Medicine, 37(1), 199–210. Available from: 10.1177/0363546508323746 18940931

[27] Safran, M.R. & Harner, C.D. (1996) Technical considerations of revision anterior cruciate ligament surgery. Clinical Orthopaedics and Related Research, 325, 50–64. Available from: 10.1097/00003086-199604000-00007 8998899

[28] Shen, X. , Qin, Y. , Zuo, J. , Liu, T. & Xiao, J. (2021) A systematic review of risk factors for anterior cruciate ligament reconstruction failure. International Journal of Sports Medicine, 42(8), 682–693. Available from: 10.1055/a-1393-6282 33784786

[29] Sonnery‐Cottet, B. , Archbold, P. , Cucurulo, T. , Fayard, J.‐M. , Bortolletto, J. , Thaunat, M. et al. (2011) The influence of the tibial slope and the size of the intercondylar notch on rupture of the anterior cruciate ligament. The Journal of Bone and Joint Surgery. British Volume, 93–B(11), 1475–1478. Available from: 10.1302/0301-620X.93B11.26905 22058297

[30] Tanaka, M.J. , Jones, K.J. , Gargiulo, A.M. , Delos, D. , Wickiewicz, T.L. , Potter, H.G. et al. (2013) Passive anterior tibial subluxation in anterior cruciate ligament‐deficient knees. The American Journal of Sports Medicine, 41(10), 2347–2352. Available from: 10.1177/0363546513498995 23928320

[31] Tanaka, Y. , Kita, K. , Takao, R. , Amano, H. , Uchida, R. , Shiozaki, Y. et al. (2018) Chronicity of anterior cruciate ligament deficiency, part 1: effects on the tibiofemoral relationship before and immediately after anatomic ACL reconstruction with autologous hamstring grafts. Orthopaedic Journal of Sports Medicine, 6(1), 2325967117750813. Available from: 10.1177/2325967117750813 29383322 PMC5784495

[32] Tanaka, Y. , Kita, K. , Takao, R. , Amano, H. , Uchida, R. , Shiozaki, Y. et al. (2018) Chronicity of anterior cruciate ligament deficiency, part 2: radiographic predictors of early graft failure. Orthopaedic Journal of Sports Medicine, 6(2), 2325967117751915. Available from: 10.1177/2325967117751915 29479543 PMC5818097

[33] Torzilli, P.A. , Xianghua Deng, Y. & Warren, R.F. (1994) The effect of joint‐compressive load and quadriceps muscle force on knee motion in the intact and anterior cruciate ligament‐sectioned knee. The American Journal of Sports Medicine, 22(1), 105–112. Available from: 10.1177/036354659402200117 8129092

[34] Webb, J.M. , Salmon, L.J. , Leclerc, E. , Pinczewski, L.A. & Roe, J.P. (2013) Posterior tibial slope and further anterior cruciate ligament injuries in the anterior cruciate ligament‐reconstructed patient. The American Journal of Sports Medicine, 41(12), 2800–2804. Available from: 10.1177/0363546513503288 24036571

[35] Webster, K.E. , Feller, J.A. , Kimp, A.J. & Whitehead, T.S. (2018) Revision anterior cruciate ligament reconstruction outcomes in younger patients: medial meniscal pathology and high rates of return to sport are associated with third ACL injuries. The American Journal of Sports Medicine, 46(5), 1137–1142. Available from: 10.1177/0363546517751141 29382207

[36] Wright, R.W. , Magnussen, R.A. , Dunn, W.R. & Spindler, K.P. (2011) Ipsilateral graft and contralateral ACL rupture at five years or more following ACL reconstruction: a systematic review. The Journal of Bone & Joint Surgery, 93(12), 1159–1165. Available from: 10.2106/JBJS.J.00898 21776554 PMC3110421

[37] Wright, R.W. , Dunn, W.R. , Amendola, A. , Andrish, J.T. , Bergfeld, J. , Kaeding, C.C. et al. (2007) Risk of tearing the intact anterior cruciate ligament in the contralateral knee and rupturing the anterior cruciate ligament graft during the first 2 years after anterior cruciate ligament reconstruction: a prospective MOON cohort study. The American Journal of Sports Medicine, 35(7), 1131–1134. Available from: 10.1177/0363546507301318 17452511

[38] Wright, R.W. , Gill, C.S. , Chen, L. , Brophy, R.H. , Matava, M.J. , Smith, M.V. et al. (2012) Outcome of revision anterior cruciate ligament reconstruction: a systematic review. Journal of Bone and Joint Surgery, 94(6), 531–536. Available from: 10.2106/JBJS.K.00733 PMC3298683 22438002

[39] Yabroudi, M.A. , Björnsson, H. , Lynch, A.D. , Muller, B. , Samuelsson, K. , Tarabichi, M. et al. (2016) Predictors of revision surgery after primary anterior cruciate ligament reconstruction. Orthopaedic Journal of Sports Medicine, 4(9), 2325967116666039. Available from: 10.1177/2325967116666039 27734019 PMC5042292

